# How did the COVID-19 pandemic reshape exercise-based mobile health in Korea?: A comparative big data analysis

**DOI:** 10.1097/MD.0000000000047371

**Published:** 2026-01-23

**Authors:** Sung-Un Park

**Affiliations:** a Department of Sports Science, Hwasung Medi-Science University, Hwaseong, South Korea.

**Keywords:** COVID-19, digital health, exercise, mobile health, South Korea

## Abstract

Mobile health (mHealth) applications became salient tools for health and exercise management during the COVID-19 pandemic. However, large-scale studies on this topic are lacking. Therefore, in this study, we analyzed changes in the key attributes and network structure of exercise-based mHealth in South Korea during and post the COVID-19 pandemic using big data analysis. A total of 32,115 data points collected from Naver and Google over 4 years were analyzed. Frequency analysis, term frequency–inverse document frequency analysis, and convergence of iterated correlations analysis were performed using TEXTOM 6.0 to identify the key attributes and clusters. The data were further analyzed to explore network structures and cluster relationships. The frequency and term frequency–inverse document frequency analyses revealed 30 high-order terms for the periods during and post the COVID-19 pandemic. The convergence of iterated correlations analysis revealed 4 clusters during the pandemic (digital transformation; wellness and lifestyle; mobile technology and personalization; healthcare and public health services) and 4 clusters post the pandemic (digital platforms and development; exercise and mobile management; mHealth and wellness; and healthcare and public health services). The COVID-19 pandemic accelerated the adoption and evolution of mHealth in South Korea, which may indicate a shift from crisis response toward greater integration into daily lives. Further, the pandemic led to the initiation of personalized digital health solutions in the post-pandemic era. These findings highlight the growing role of mHealth in public health and wellness and provide insights into future developments in digital health solutions.

## 1. Introduction

The COVID-19 pandemic altered lives worldwide, particularly people’s health-related behaviors and practices. In South Korea, strict social distancing measures and the temporary closure of fitness centers necessitated a shift to digital solutions for health and exercise management.^[1]^ These changes accelerated the adoption of mobile health (mHealth) and brought fundamental shifts in user behavior and service usage patterns related to exercise and wellness.^[2]^

MHealth applications became essential tools during the COVID-19 pandemic, as they increased health awareness among the public.^[3]^ Today, they serve as a novel way to develop and evaluate public health interventions. However, Yang et al^[4]^ have reported a lack of large-scale systematic evaluation of these applications. Additionally, existing research has highlighted the need to examine the sustainability and scalability of mHealth applications in enhancing public health outcomes.

Research on exercise-based mHealth during the pandemic primarily focused on patient-focused interventions.^[5,6]^ Sporrel et al^[7]^ conducted a systematic literature review of exercise-based mHealth. However, the limitations of the previous studies, such as varying research quality, heterogeneous intervention designs, potential publication bias, and rapid obsolescence of findings owing to technological advances, highlight the need for more robust research.

According to the Physical Activity Guidelines for Americans, adults (including those with chronic conditions) should engage in 150 to 300 minutes of moderate–intensity physical activity weekly.^[8]^ Exercising is one of the most effective ways to increase 1’s physical activity.^[9,10]^ Advances in mHealth technology, such as mobile applications and wearable devices, have enabled real-time collection and provision of exercise data.^[11]^ Using these technologies, users can monitor their exercise data and health status. For instance, wearable devices such as smartwatches can track various fitness metrics, such as sweat secretion, respiration rate, muscle tension, and body temperature, providing users with valuable healthcare information.^[12]^

Despite these advancements, research on exercise-based mHealth is limited by methodological issues, participant diversity, technical challenges, inconsistent outcome measures, privacy concerns, economic barriers, and regulatory and ethical considerations.^[13]^ These limitations highlight the need for alternative research approaches. Previous studies on mHealth had limitations such as restricted scalability, methodological shortcomings, and insufficient participant diversity. The distinctive properties of mHealth data correspond to the well-known dimensions of big data: volume, variety, velocity, veracity, and value. mHealth platforms, such as those developed by Apple and Garmin, exemplify these characteristics by generating continuous streams of physiological and behavioral data (including activity levels, sleep quality, and energy expenditure) that can be analyzed in real time.^[14]^ Such real-time and large-scale data streams demonstrate how emerging big data technologies can overcome past research barriers by enabling broader analysis, providing timely insights, and incorporating more representative populations.^[15]^ From this perspective, big data holds significant potential to advance mHealth, not only in medical and public health research but also in chronic disease monitoring, preventive care, emergency response, and reduction of healthcare expenditures.^[15]^ Comprehensive research on mHealth is crucial for understanding the evolving needs and behaviors of users in a digital environment. Therefore, in this study, we aimed to provide insight into the sustainability and scalability of mHealth technologies in improving public health outcomes.^[16,17]^

Big data refers to vast and complex datasets that cannot be processed using traditional data management techniques.^[18]^ It has been widely used for examining social awareness and behavioral intentions and for predicting trends.^[19]^ Furthermore, it has been extensively beneficial in fields such as healthcare, medical research, and public health.^[20,21]^ With an advanced network infrastructure, South Korea has the optimum conditions for harnessing the benefits of big data.^[22,23]^ In this context, big data can be an invaluable analytical tool in exercise-based mHealth research, helping researchers gain a precise understanding of social awareness shifts and behavioral patterns.

Therefore, in this study, we used big data analysis to investigate the key attributes and clustering patterns of exercise-based mHealth in South Korea, comparing pre- and post-COVID-19 pandemic trends. The comparative analysis aimed to enhance mHealth by offering more personalized and effective exercise solutions. Furthermore, ensuring that exercise-based mHealth research is accessible to diverse populations is critical for improved public health outcomes. By leveraging big data, we specifically explored the impact of the COVID-19 pandemic on the adoption and use of exercise-based mHealth South Korea.

## 2. Methods

### 2.1. Ethical considerations

This study was approved by the Public Institutional Review Board, Republic of Korea (No. P01-202404-01-066).

### 2.2. Study design

We employed big data analysis to investigate changes in the key attributes and network structure of exercise-based mHealth pre- and post-COVID-19 pandemic period in Korea. Using TEXTOM (The Imc Inc., Daegu, Korea), large datasets of text were analyzed to identify trends, perceptions, key attributes, and clusters concerning pre- and post-COVID-19 pandemic mHealth. TEXTOM is a user-friendly analysis package that has adapted the full-text software developed by Professor Loet Leydesdorff for Korean text analysis.^[24]^ To address the unequal time spans, frequency data were exported into Microsoft Excel (Microsoft Corp., Redmond) for monthly normalization and visualization. Subsequently, UCINET 6 (Analytic Technologies, Lexington) was used to perform convergence of iterated correlations (CONCOR) analysis to explore structural relationships and clusters.

### 2.3. Data collection

TEXTOM version 6.0 was used to collect unstructured textual data from Naver and Google. Data were collected from Naver and Google because they are the 2 most widely used online platforms in Korea.^[25]^ To maintain a targeted focus, the keywords “exercise + mobile + health” were used. In addition, because the analysis was based on Korean texts, an equivalent Korean query “운동+모바일+헬스” was applied to both Naver and Google. These search words were selected to ensure a comprehensive coverage of relevant pre- and post-COVID-19 pandemic discourse and to enhance the reproducibility of the data collection process. To collect data pertaining to the period during the COVID-19 pandemic, the data collection period was from March 1, 2020 (when COVID-19 was officially declared in Korea) to May 31, 2023 (when COVID-19 transitioned to endemic status). To collect the post-pandemic data, data collection period extended from June 1, 2023, to May 31, 2024. The post-pandemic period was considered as the period up to December 2024 due to data availability constraints. To minimize distortions caused by the unequal lengths of the “during pandemic” (39 months) and “post-pandemic” (12 months) periods, keyword frequencies were normalized by dividing the total counts by the number of months in each period, thereby yielding monthly averages. The collected unstructured data underwent a purification process in which 1-syllable Korean words (typically meaningless in context) as well as common functional stop-words such as particles and conjunctions were removed, duplicate or synonymous terms were consolidated, and special characters and numbers without analytical value were excluded. Term frequency–inverse document frequency (TF–IDF) weighting was conducted using the default algorithm provided in TEXTOM. Table 1 presents the characteristics of data collection.

**Table 1:** Characteristics of data collection.

Characteristic	Description
Language	Korean
Tool	TEXTOM (http://textom.co.kr)
Online platforms	Naver, Google
Keywords	Exercise, Mobile, Health
Period	March 1, 2020, to May 31, 2023 (During the COVID-19 pandemic)
	June 1, 2023, to May 31, 2024 (After the COVID-19 pandemic)

*Note:* The keywords “exercise + mobile + health” (and their Korean equivalents *“운동 + 모바일 + 헬스”*) were selected to capture the discourse specifically related to exercise-based mHealth. This rationale aligns with the study’s objective of examining how digital health tools were discussed in relation to physical activity and health management during and after the COVID-19 pandemic.

### 2.4. Data analysis

Text mining analyses were conducted to assess changes in the perceptions of exercise-based mHealth during and post the COVID-19 pandemic. Text mining involves analyzing large volumes of text to extract patterns and relationships and can reveal meaningful insights and trends.^[26]^ Frequency and TF–IDF analyses were conducted as part of text mining to identify the key terms and their importance in the dataset. Frequency analysis counts the occurrence of specific terms within a dataset and provides an overview of prominent topics in exercise-based mHealth. Then, the TF–IDF analysis calculates the weight of each term and reveals terms that are particularly distinctive within the corpus. This analysis helps identify the most relevant terms beyond common phrases, adding depth to the analysis.

In this study, frequency and TF–IDF matrices were generated using TEXTOM. To account for the unequal time spans between the 2 periods, the extracted frequency data were exported to Microsoft Excel, where monthly normalized values were manually calculated and visualized. TF–IDF results were then reviewed to identify the top-ranked keywords for each period, focusing on terms with relatively high weights compared with their raw frequency, thereby highlighting period-specific characteristics of mHealth discourse.

After text mining analyses, a CONCOR analysis was performed to identify potential clusters within the data and examine the relationships between the key terms. This analysis identifies patterns in the relationships between terms and groups the terms based on their structural equivalence. The higher the similarity in relational patterns, the greater the degree of structural equivalence between terms. The CONCOR analysis groups terms based on similar relational patterns, which reveals thematic clusters within the data. This analysis helps 1 map time-based shifts in the discourse being investigated.^[27]^ Each cluster was named through comprehensive review and interpretation processes. This yielded themes reflecting the complexities of mHealth discourse during and post the COVID-19 pandemic. In this study, the processed matrices were imported into UCINET 6, and CONCOR was applied through repeated correlation procedures until convergence was achieved. The optimal number of clusters was determined through iterative examination of the correlation matrix and visual analysis of the dendrogram structure. To ensure convergence stability, iterative calculations were conducted with a maximum of 25 iterations, using the Pearson correlation coefficient as a similarity index, and binary partitioning was repeated twice (n = 2). Meaningfully connected keyword groups were identified through this process, and a 4-cluster solution was selected because it exhibited stable convergence and clear separation between clusters and minimized unnecessary fragmentation. First, each cluster was algorithmically derived through CONCOR analysis, which grouped terms according to structural equivalence. Subsequently, the naming of clusters was refined through a systematic review of the included keywords, guided by contextual interpretation and theoretical relevance. In addition, the proposed cluster names were subjected to independent evaluation by 2 professors with established related research expertise. This combined approach ensured that the labeling was not based solely on subjective interpretation but also on algorithmic clustering results.

## 3. Results

### 3.1. Data collection

Table 2 shows the results of data collection. The collection of data pertaining to the period during the pandemic produced 20,829 data points with a volume of 10,217.88 KB. The collection of data pertaining to the post-pandemic period produced 11,286 data points with a volume of 5599.03 KB. In total, 32,115 data points and 15,816.91 KB were collected using TEXTOM.

**Table 2:** Results of data collection.

Data collected/period		During the COVID-19 pandemic (Average per month)	After the COVID-19 pandemic (average per month)	Total
Type of data	Source			
Number of data points	Naver	20,227 (828.7)	10,943 (911.9)	31,170
	Google	602 (15.4)	343 (28.6)	945
	Total	20,829 (844.1)	11,286 (940.5)	32,115
Volume	Naver	10,007.13 KB	5485.59 KB	15,492.72 KB
	Google	210.75 KB	113.44 KB	324.19 KB
	Total	10,217.88 KB	5599.03 KB	15,816.91 KB

*Note*: Monthly averages were calculated by dividing the total number of data points by the number of months in each period (during 39 months; after 12 months), as TEXTOM does not provide month-by-month data.

### 3.2. Frequency analysis

**Figure 1.**
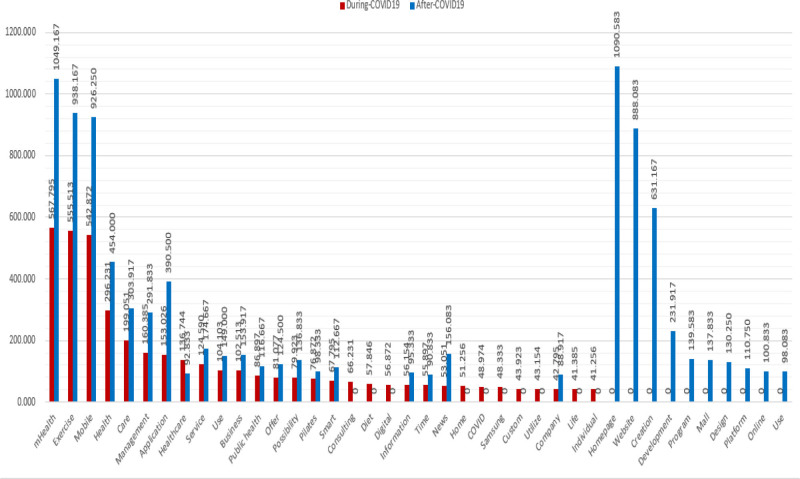
Monthly normalization of term frequencies to ensure comparability between the 2 periods. Red bars represent keyword frequencies during the COVID-19 pandemic, and blue bars represent frequencies in the post-pandemic era.

Table 3 summarizes the frequency analysis and monthly average frequencies of terms related to mHealth-based exercise during and post the COVID-19 pandemic. To ensure valid comparisons between the 2 periods, frequencies were normalized on a monthly basis (Fig. 1). The top 10 most frequently occurring terms during the pandemic were mHealth (22,144; 567.795), exercise (21,665; 555.513), mobile (21,172; 542.872), health (11,553; 296.231), care (7763; 199.051), management (6255; 160.385), application (5968; 153.026), healthcare (5333; 136.744), service (4859; 124.590), and use (4060; 104.103).

**Table 3:** Top 30 most frequently occurring terms identified by frequency analysis.

	During the COVID-19 pandemic		Normalized frequency per month	After the COVID-19 pandemic		Normalized frequency per month
	Term	Frequency		Term	Frequency	
1	mHealth	22,144	567.795	Homepage	13,087	1090.583
2	Exercise	21,665	555.513	mHealth	12,590	1049.167
3	Mobile	21,172	542.872	Exercise	11,258	938.167
4	Health	11,553	296.231	Mobile	11,115	926.250
5	Care	7763	199.051	Website	10,657	888.083
6	Management	6255	160.385	Creation	7574	631.167
7	Application	5968	153.026	Health	5448	454.000
8	Healthcare	5333	136.744	Application	4686	390.500
9	Service	4859	124.590	Care	3647	303.917
10	Use	4060	104.103	Management	3502	291.833
11	Business	3998	102.513	Development	2783	231.917
12	Public health	3389	86.897	Service	2096	174.667
13	Offer	3162	81.077	News	1873	156.083
14	Possibility	3117	79.923	Business	1847	153.917
15	Pilates	2998	76.872	Time	1788	149.000
16	Smart	2644	67.795	Program	1675	139.583
17	Consulting	2583	66.231	Mall	1654	137.833
18	Diet	2256	57.846	Possibility	1642	136.833
19	Digital	2218	56.872	Design	1563	130.250
20	Information	2190	56.154	Offer	1494	124.500
21	Time	2180	55.897	Public health	1400	116.667
22	News	2069	53.051	Smart	1352	112.667
23	Home	1999	51.256	Platform	1329	110.750
24	COVID	1910	48.974	Online	1210	100.833
25	Samsung	1885	48.333	Pilates	1180	98.333
26	Custom	1713	43.923	Use	1177	98.083
27	Utilize	1683	43.154	Information	1144	95.333
28	Company	1669	42.795	Healthcare	1114	92.833
29	Life	1614	41.385	Solution	1090	90.833
30	Individual	1609	41.256	Company	1067	88.917

*Note*: Normalized frequency per month was calculated by dividing the total frequency of each term by the number of months in each period (during 39 months; after 12 months), as TEXTOM does not provide month-by-month data.

The top 10 most frequently occurring post-pandemic terms were homepage (13,087; 1090.583), mHealth (12,590; 1049.167), exercise (11,258; 938.167), mobile (11,115; 926.250), website (10,657; 888.083), creation (7574; 631.167), health (5448; 454.000), application (4686; 390.500), care (3647; 303.917), and management (3502; 291.833).

### 3.3. TF–IDF analysis

Table 4 summarizes the results of the TF–IDF analysis of terms related to mHealth-based exercise during and post the COVID-19 pandemic. The top 10 most relevant terms during the pandemic were health (14,671), healthcare (11,440), management (9913), care (9787), application (9093), service (8445), business (8337), Pilates (8056), public health (7683), and use (7331). The top 10 most relevant post-pandemic terms were homepage (25,075), website (20,386), creation (15,931), health (7313), application (5723), development (5600), care (5216), management (5181), news (4388), and business (4189).

**Table 4:** Top 30 most relevant terms identified by TF–IDF analysis.

	During the COVID-19 pandemic		After the COVID-19 pandemic	
	Term	TF–IDF	Term	TF–IDF
1	Health	14,671	Homepage	25,075
2	Healthcare	11,440	Website	20,386
3	Management	9913	Creation	15,931
4	Care	9787	Health	7313
5	Application	9093	Application	5723
6	Service	8445	Development	5600
7	Business	8337	Care	5216
8	Pilates	8056	Management	5181
9	Public health	7683	News	4388
10	Use	7331	Business	4189
11	Offer	6359	Mall	4036
12	Possibility	6337	Service	4014
13	Smart	6162	Time	3958
14	Consulting	6055	Design	3762
15	Diet	6012	Sanbon city	3708
16	mHealth	5786	Program	3639
17	Digital	5693	Public health	3543
18	Information	5384	Pilates	3409
19	Home	5361	Possibility	3347
20	News	5320	Smart	3226
21	Samsung	5264	Offer	3191
22	Time	5223	Gym	3101
23	COVID	4874	Platform	3059
24	Exercise	4863	mHealth	2956
25	Watch	4732	Online	2937
26	Body	4583	Use	2889
27	Company	4550	Solution	2791
28	Gym	4511	Information	2788
29	Custom	4362	Exercise	2786
30	Reservation	4356	Company	2773

*Note*: A relatively high TF–IDF score indicates that a term is not only used frequently but also particularly distinctive within the corpus, thereby highlighting its unique importance in characterizing each period.

TF–IDF = term frequency–inverse document frequency.

### 3.4. CONCOR analysis

**Figure 2.**
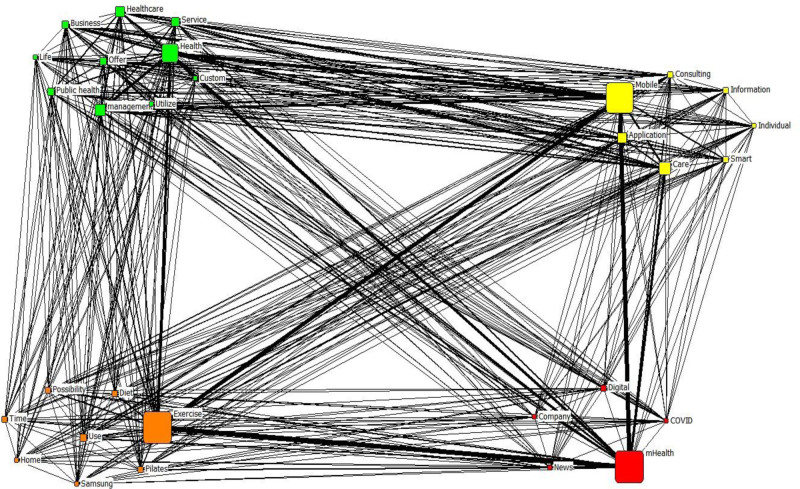
Results of the CONCOR analysis for the period during the COVID-19 pandemic. Colors indicate the 4 thematic clusters: red: digital transformation; orange: wellness and lifestyle; yellow: mobile technology and personalization; green: healthcare and public health services. CONCOR = convergence of iterated correlations.

The CONCOR analysis was performed to classify homogeneous groups based on the relevance of the correlations in the network. Table 5 presents its results for the period during the COVID-19 pandemic. Evidently, 4 clusters were obtained: digital transformation; wellness and lifestyle; mobile technology and personalization; and healthcare and public health services. The terms “mHealth,” “digital,” “news,” “COVID-19,” and “company” were grouped as “digital transformation.” The terms “exercise,” “usage,” “possibility,” “Pilates,” “diet,” “time,” “home,” and “Samsung” were grouped as “wellness and lifestyle.” The terms “mobile,” “care,” “application,” “smart,” “consulting,” “information,” and “individual” were grouped as “mobile technology and personalization.” Finally, the terms “health,” “management,” “healthcare,” “service,” “business,” “public health,” “offer,” “custom,” “utilize,” and “life” were grouped as “healthcare and public health services.” Figure 2 illustrates the derived clusters.

**Table 5:** Results of CONCOR analysis for the period during the COVID-19 pandemic.

	Cluster (numbers of term)	Terms (degree centrality)	Density (binary)
			Weighted average Tie strength
1	Digital transformation (5)	mHealth (0.219), digital (0.025), news (0.013), COVID-19 (0.020), company (0.014)	1.0
			1010.0
2	Wellness and lifestyle (8)	Exercise (0.198), usage (0.043), possibility (0.031), Pilates (0.022), diet (0.024), time (0.022), home (0.020), Samsung (0.020)	1.0
			1239.64
3	Mobile technology and personalization (7)	Mobile (0.238), care (0.117), application (0.083), smart (0.039), consulting (0.039), information (0.028), individual (0.024)	1.0
			2270.14
4	Healthcare and public health services (10)	Health (0.189), management (0.106), healthcare (0.078), service (0.077), business (0.073), public health (0.067), offer (0.057), custom (0.036), utilize (0.027), life (0.023)	1.0
			2656.69

*Note:* Binary density values (0–1) indicate whether clusters form fully connected subnetworks. In this dataset, all clusters show complete connectivity (density = 1.0). To capture differences in cluster cohesion, weighted densities (average tie strength based on co-occurrence frequency) were additionally reported.

CONCOR = convergence of iterated correlations.

**Figure 3.**
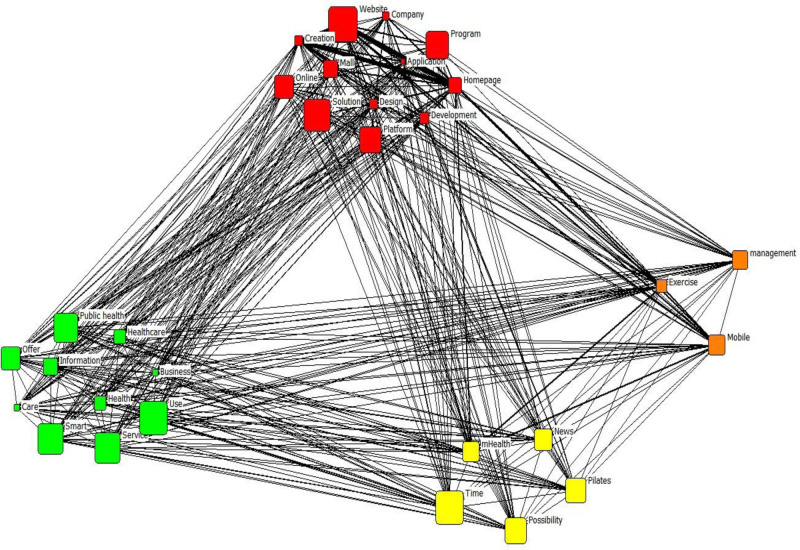
Results of the CONCOR analysis for the period after the COVID-19 pandemic. Colors indicate the 4 thematic clusters identified: red: digital platforms and development; orange: exercise and mobile management; yellow: mHealth and wellness; green: healthcare and public health services. CONCOR = convergence of iterated correlations.

Table 6 presents the results of the CONCOR analysis for the post-COVID-19 pandemic period. Four clusters were derived: digital platforms and development; exercise and mobile management; mHealth and wellness; and healthcare and public health services. The terms “homepage,” “website,” “creation,” “application,” “development,” “program,” “mall,” “design,” “platform,” “online,” “solution,” and “company” were grouped as “digital platforms and development.” The terms “exercise,” “mobile,” and “management” were grouped as “exercise and mobile management.” The terms “mHealth,” “news,” “time,” “possibility,” and “Pilates” were grouped as “mHealth and wellness.” The terms “health,” “care,” “service,” “business,” “offer,” “public health,” “smart,” “usage,” “information,” and “healthcare” were grouped as “healthcare and public health services.” Figure 3 illustrates the derived clusters.

**Table 6:** Results of CONCOR analysis for the period after the COVID-19 pandemic.

	Cluster (numbers of term)	Terms (degree centrality)	Density (binary)
			Weighted average tie strength
1	Digital platforms and development (12)	Homepage (0.118), website (0.102), creation (0.080), application (0.031), development (0.029), program (0.015), mall (0.020), design (0.017), platform (0.011), online (0.011), solution (0.010), company (0.012)	1.0
			8681.18
2	Exercise and mobile management (3)	Exercise (0.034), mobile (0.044), management (0.018)	1.0
			6803.33
3	mHealth and wellness (5)	mHealth (0.039), news (0.003), time (0.003), possibility (0.004), Pilates (0.003)	1.0
			1007.60
4	Healthcare and public health services (10)	Health (0.018), care (0.012), service (0.009), business (0.008), offer (0.006), public health (0.006), smart (0.005), usage (0.003), information (0.004), healthcare (0.004)	1.0
			978.80

*Note*: Binary density values (0–1) indicate whether clusters form fully connected subnetworks. In this dataset, all clusters show complete connectivity (density = 1.0). To capture differences in cluster cohesion, weighted densities (average tie strength based on co-occurrence frequency) were additionally reported.

CONCOR = convergence of iterated correlations.

In Tables 5 and 6, the degree centrality of each keyword represents the extent to which a term is directly connected to other terms within the semantic network. Higher centrality scores indicate that a keyword functions as a key node, exerting greater influence on the structure and meaning of the discourse.

## 4. Discussion

### 4.1. Frequency analysis

As remote environments became the new normal, managing exercise and health using mHealth technologies and applications became essential during the COVID-19 pandemic. This shift is reflected in the frequent occurrence of terms such as “mHealth,” “exercise,” “mobile,” “health,” “care,” “management,” “application,” “healthcare,” “service,” and “use.”

The term “mHealth” refers to health management and service delivery through mobile technology. Pandemic-induced restrictions on outdoor activities and closures of fitness facilities coincided with an increase in the digitalization of exercise routines.^[28,29]^ People increasingly used mHealth apps to monitor their health, log exercise, and communicate remotely with healthcare professionals to reduce in-person visits.^[30]^ This could be the reason for the frequent co-occurrence of the terms such as “exercise” and “mobile” during the COVID-19 pandemic.

The frequent occurrence of terms like “health,” “care,” and “management” suggest that mHealth technologies complemented traditional healthcare systems during the pandemic. Amid movement restrictions and limited access to traditional medical facilities, mHealth technologies provided a new way to manage health conditions and access healthcare services.^[31]^

The pandemic increased the need to take a proactive approach to exercise and health management. This led to the rise of “applications” that helped meet such needs. These applications offered diverse “services” related to “healthcare,” enhancing user convenience and enabling effective health management in remote environments. Consequently, “use” was a frequently occurring term, reflecting the broad acceptance of mHealth apps during the COVID-19 pandemic.

Overall, the COVID-19 pandemic accelerated the development of mHealth and mHealth-based solutions for exercise, making them essential components of modern health management, a transition from their previous use as optional tools. This trend is expected to continue post-pandemic, as mHealth technologies are expected to further integrate into daily lives.

However, post-COVID-19 pandemic, terms such as “homepage,” “website,” “creation,” “mHealth,” “exercise,” “mobile,” “health,” “care,” and “management” became prominent. The increased frequency of terms such as “homepage” and “website” reflects a shift toward more established digital platforms and online spaces for health management and exercise routines.^[32]^ Similarly, the heightened appearance of the term “creation” suggests a growing interest in user-generated content and personalized health and exercise plans.^[33]^ Taken together, these patterns may suggest that users are becoming more familiar with digital health tools and may be moving toward more interactive and personalized solutions; however, such interpretations should be approached with caution, as keyword frequency alone cannot directly confirm user behavior.

A comparison of frequently occurring terms during and post-pandemic reveals a shift in the usage of mHealth, transcending from its use as a crisis response to its more integrated use in daily lives.^[34]^ During the pandemic, the primary focus was on adapting quickly to disruptions in traditional health and exercise routines, as shown by the prominence of terms related to immediate use, management, and service. In contrast, the post-pandemic period shows an emphasis on the integration and refinement of these digital tools, with increased interest in established online platforms (“homepage” and “website”) and personalized content creation. This shift suggests that pandemic’s diminishing impact was followed by growing attention to the quality and relevance of digital health tools among both users and service providers, though further evidence would be required to substantiate behavioral intent.

### 4.2. TF–IDF analysis

During the COVID-19 pandemic, the most relevant terms were “health,” “healthcare,” “management,” “care,” “application,” “service,” “business,” “Pilates,” “public health,” and “use.” These terms reflect specific healthcare trends and new opportunities brought about by the pandemic.

The emergence of terms like “business” and “Pilates” signifies new business opportunities as the healthcare and fitness industries rapidly digitalized themselves during the pandemic. With the closure of physical spaces, such as gyms and Pilates studios,^[29]^ many service providers transitioned to digital platforms,^[28]^ offering remote exercise programs and personalized workout solutions. Pilates, in particular, gained popularity as an easy-to-follow home exercise.^[35]^ It allowed mHealth applications to provide personalized exercise management services. This shift may reflect how business organizations appeared to adapt and evolve during the pandemic.

The prominence of the term “public health” underscores its heightened importance during the pandemic. mHealth applications became essential tools for protecting individual and community health during the pandemic.^[36]^ They provided various public health services, including self-monitoring, information on preventive measures, and access to medical consultations, all aimed at curbing virus transmission.

However, post the COVID-19 pandemic, the most relevant terms shifted to “homepage,” “website,” “creation,” “health,” “application,” “development,” “care,” “management,” “news,” and “business.” This change reflects a shift in the focus from immediate health-crisis management to a focus on digital presence, content creation, and the development of long-term health solutions. In other words, it highlights the acceleration of digitalization in health communication and service delivery.^[37]^ It also shows that as the COVID-19 crisis eases, there is a movement toward building sustainable digital health solutions.^[38]^ Consequently, “business” suggests a transition from pandemic survival strategies to strategies aimed at stabilization and growth.^[39]^

The transition in relevant terms from the pandemic to the post-pandemic period demonstrates a clear shift in the perception of mHealth: from being perceived as a crisis response measure to being perceived as a more strategic and sustainable approach to mHealth. During the pandemic, the main focus was addressing health management and healthcare needs, adopting digital solutions, and maintaining physical activity amid social distancing. In contrast, the post-pandemic period shows an emphasis on the integration of digital health platforms (“homepage” and “website”) and the development of new tools and services (“creation” and “development”). This shift reflects how innovations and adaptations made during the pandemic are being turned into long-term health solutions.

### 4.3. CONCOR analysis

During the pandemic, the 1st cluster shows that the discourse focused on the digital transformation of healthcare (“mHealth”), media (“news”), and business operations. In particular, healthcare digitalization was frequently highlighted, suggesting that the crisis coincided with increased attention to digital solutions.^[37]^ As people realized the importance of remote health management, mHealth applications for exercise management gained popularity. Advancements in digital technology popularized health management and personalized exercise services (through smartphones and wearable devices), while media coverage heightened public interest. Additionally, healthcare companies offered innovative mHealth solutions for health and exercise management. In summary, the COVID-19 pandemic accelerated the spread of digital healthcare and mHealth services.

The 2nd cluster highlights the significant increase in the use of mHealth applications in daily life during the COVID-19 pandemic as people sought ways to exercise at home. Pilates and dieting emerged as major fitness trends with the growing use of programs and applications for home workouts. Time management also became important, as many people aimed to allocate time for exercise while working from home. Additionally, keywords related to Samsung suggest that large corporations were mentioned in the discourse around mHealth, possibly reflecting their role in providing devices and platforms that supported home workout experiences.^[40]^ The co-occurrence of terms related to mHealth and home exercise may indicate that such applications were increasingly associated with personal health and exercise management during the pandemic, although keyword data cannot directly confirm actual user behavior.

The 3rd cluster demonstrates changes in how people managed their health using mobile devices during the pandemic. There was a surge in the use of mobile applications for personalized exercise programs. These applications allowed users to access smart health information and develop personalized exercise and health management plans. They also provided exercise consulting services, enabling users to receive expert advice and manage their health and exercise routines more systematically.^[41]^ This trend indicates the expansion of remote health management and exercise services during the pandemic and the growing demand for personalized mobile technology-based healthcare solutions.

The 4th cluster indicates that mobile technology-based healthcare services expanded remotely during the pandemic, providing customized health management solutions. In particular, mHealth technologies played a crucial role in public health by providing essential information and supporting health management. Moreover, they enhanced healthcare accessibility, aided efficient resource allocation, and reduced the burden on healthcare systems.^[42]^ As a result, mHealth became an important tool to manage health and played a key role in protecting and maintaining public health during the pandemic.

The 1st cluster shows that the mHealth exercise industry has grown rapidly around digital platforms and applications in the post COVID-19 pandemic period. Creating and designing homepages and websites have become essential for mHealth companies to improve user accessibility. Applications that provide exercise management, health data analysis, and personalized healthcare services have become widely popular with the development of exercise programs and solutions.^[43]^ Moreover, online healthcare platforms now offer “malls” where users can purchase exercise equipment and related products, making it easier for them to access healthcare products and services. Consequently, the mHealth industry has expanded further in the online environment in the post-COVID-19 pandemic period. Companies are opening new avenues in healthcare and exercise management by pursuing innovation and improvements in digital platforms.

The 2nd cluster highlights changes in how exercise is managed through mHealth technology in the post-pandemic period. MHealth applications have become an essential tool to track and manage health, as they offer various features to record and analyze exercise routines.^[44]^ Consequently, in the post-pandemic period, the frequent association of mHealth with exercise management may suggest an increasing discourse around its centrality, though this does not directly establish behavioral transformation.

The 3rd cluster indicates that mHealth technologies have become a crucial tool for exercise and health management in the post-pandemic period. With the increasing demand for remote exercise and health management, mHealth-related solutions and programs are frequently covered in news media, increasing people’s awareness of the potential of these technologies. This has opened up more flexible exercise options, particularly Pilates.^[45]^ Consequently, post-pandemic mHealth technology has enabled exercise without time and location constraints, with Pilates garnering significant public interest.

The 4th cluster demonstrates that mHealth technology has become essential not only for individual health management but also for public health management. Today, remote healthcare services are available, and there is a growing demand for personalized health management solutions. There is widespread use of smart technology to collect and analyze health data in real time, making health information more accessible to users. In the public health sector, mHealth has strengthened preventive and management services by providing essential health information to the public.^[46]^ Simultaneously, healthcare-related businesses have grown around digital platforms, with various companies offering mHealth applications that provide personalized health management and exercise programs. As a result, smart technology use has expanded in the healthcare and public health fields, and business opportunities have increased in the field of health management.

Despite the valuable insights this study provides into the role and potential of mHealth technology, this study has some limitations. First, the study collected and analyzed big data from specific online platforms in Korea (Naver and Google), limiting the representativeness and diversity of the data and the findings. Thus, the results may not be generalizable to regions outside Korea or other cultural contexts. Further, notably, these findings are derived from Korean online platforms (Naver and Google) and thus may reflect platform-specific and cultural discourse, which could further limit the generalizability of the results. Second, because of the nature of text mining, it may not have fully captured the semantic relationships or contexts between keywords. As text mining analyzes patterns based on the frequency and weight of terms, it has limitations in identifying deep semantic connections between keywords. Another limitation is that the TF–IDF analysis, inherently, does not capture the semantic context of words, which may result in the partial loss of nuanced meanings. However, to mitigate this limitation, we complemented the TF–IDF results with CONCOR analysis and comprehensive interpretive review to preserve thematic coherence. Third, the clusters derived through the CONCOR analysis were named according to the interpretation of the researchers, which means that the interpretation of the results can differ based on subjective perspectives. However, by examining the evolving landscape of exercise-based mHealth, this study offers critical insights for enhancing the efficacy and appeal for digital health interventions.

These findings may offer useful guidance for future design and policy development regarding mHealth in Korea. The results of this study help identify user preferences, discourse dynamics, and emerging roles of mHealth technologies, which could help inform strategies to improve accessibility, personalization, and service integration within the healthcare system. While the present study does not allow for deterministic conclusions, its findings indicate potential possibilities that can guide policymakers and developers to plan and implement appropriate mHealth initiatives.

## 5. Conclusion

Exercise-based mHealth underscored the significance of healthcare, technology, and wellness both during and post the COVID-19 pandemic period. However, the scope and applications of mHealth continue to evolve. The post-pandemic period marks a clear shift towards more specialized and technology-driven solutions, reflecting rapid technological advancements and societal changes prompted by the pandemic. The pandemic acted as a catalyst for change, accelerated digital adoption, and emphasized the importance of health and well-being, thus promoting more targeted and sophisticated approaches in mHealth.

These developments may suggest that in the future, smart technology and data-driven personalized solutions could take on a central role in the mHealth field. Consequently, exercise, health, and wellness management may be increasingly provided via mHealth in a more goal-oriented and efficient manner, which may contribute to improved public health.

The findings of this study contribute to the existing literature on mHealth adoption during global health crises and provide valuable insights for stakeholders seeking to optimize digital health solutions in the post-pandemic era.

## Author contributions

**Conceptualization:** Sung-Un Park.

**Data curation:** Sung-Un Park.

**Formal analysis:** Sung-Un Park.

**Funding acquisition:** Sung-Un Park.

**Investigation:** Sung-Un Park.

**Methodology:** Sung-Un Park.

**Project administration:** Sung-Un Park.

**Resources:** Sung-Un Park.

**Software:** Sung-Un Park.

**Supervision:** Sung-Un Park.

**Validation:** Sung-Un Park.

**Visualization:** Sung-Un Park.

**Writing – original draft:** Sung-Un Park.

**Writing – review & editing:** Sung-Un Park.
